# Sodium channel Na_v_1.7 immunoreactivity in painful human dental pulp and burning mouth syndrome

**DOI:** 10.1186/1471-2202-11-71

**Published:** 2010-06-08

**Authors:** Kiran Beneng, Tara Renton, Zehra Yilmaz, Yiangos Yiangou, Praveen Anand

**Affiliations:** 1 Dental Institute, King's College London, Guy's Hospital, Oral Surgery Department, Great Maze Pond, London, UK; 2 Peripheral Neuropathy Unit, Hammersmith Hospital, Imperial College London, London, UK

## Abstract

**Background:**

Voltage gated sodium channels Na_v_1.7 are involved in nociceptor nerve action potentials and are known to affect pain sensitivity in clinical genetic disorders.

**Aims and Objectives:**

To study Na_v_1.7 levels in dental pulpitis pain, an inflammatory condition, and burning mouth syndrome (BMS), considered a neuropathic orofacial pain disorder.

**Methods:**

Two groups of patients were recruited for this study. One group consisted of patients with dental pulpitis pain (n = 5) and controls (n = 12), and the other patients with BMS (n = 7) and controls (n = 10). BMS patients were diagnosed according to the International Association for the Study of Pain criteria; a pain history was collected, including the visual analogue scale (VAS). Immunohistochemistry with visual intensity and computer image analysis were used to evaluate levels of Na_v_1.7 in dental pulp tissue samples from the dental pulpitis group, and tongue biopsies from the BMS group.

**Results:**

There was a significantly increased visual intensity score for Na_v_1.7 in nerve fibres in the painful dental pulp specimens, compared to controls. Image analysis showed a trend for an increase of the Na_v_1.7 immunoreactive % area in the painful pulp group, but this was not statistically significant. When expressed as a ratio of the neurofilament % area, there was a strong trend for an increase of Na_v_1.7 in the painful pulp group. Na_v_1.7 immunoreactive fibres were seen in abundance in the sub-mucosal layer of tongue biopsies, with no significant difference between BMS and controls.

**Conclusion:**

Na_v_1.7 sodium channel may play a significant role in inflammatory dental pain. Clinical trials with selective Na_v_1.7 channel blockers should prioritise dental pulp pain rather than BMS.

## Background

Orofacial pain conditions are common and debilitating. Few studies have investigated the role of novel key pain ion channels, such as Na_v_1.7, in these conditions. Such studies may lead to the development of more effective treatments.

Dental pain is the most common symptom of diseased tooth pulp, often as a result of coronal caries of the tooth [1]. The mature human dental pulp is densely innervated with fibres that originate from the trigeminal ganglion [2]. The normal pulp seems insensitive to exteroceptive stimuli; however, in pathological states such as pulpitis (inflammation of the pulp), electrical, thermal, mechanical and chemical stimuli all produce a nociceptive response [3]. Primary and permanent tooth pulps contain 70-90% C-fibres, and thin myelinated A delta fibres [4]. The majority of nerve fibres terminate in the coronal region of the pulp, forming a subodontoblast plexus, with 40% terminating in the dentinal tubules close to the odontoblast processes [5]. Strong correlations have been reported between the afferent discharge frequency of human pulp nociceptors and pain levels [6].

Nociceptors within the oral mucosa have also been implicated in another orofacial pain condition, burning mouth syndrome (BMS). The International Association for the Study of Pain (IASP) defines BMS as a distinct neuropathic orofacial pain condition characterised by bilateral burning oral mucosal pain, usually affecting the anterior two thirds of the tongue with a lack of any visible signs of mucosal pathology, and of more than 6 months duration [7]. The pain intensity ranges from moderate to severe throughout the day and may last several years [8,9]. Initiation can be spontaneous or associated with systemic factors such as diabetes, nutritional deficiencies, hormonal changes, psychological disorders as well as local causes including oral infections, allergies, salivary gland dysfunction and dental treatment [10].

The underlying mechanisms involved in pain initiation and conduction in BMS and pulpitis are still not fully understood, but are likely to involve sodium ion channels. Voltage gated sodium (Na_v_) channels are known to play a key role in elicitation of action potentials in neurons, including nociceptors [11]. There are 9 sub-types of sodium (Na_v_) channels in humans, and changes in their expression may underlie hypersensitivity in pain states [12]. A subset of voltage-gated sodium channels that include Na_v_1.3, Na_v_1.7, Na_v_1.8 and Na_v_1.9, have been shown to modulate pain [4,13]. These isoforms display unique expression patterns within specific tissues [12], and are up- or down-regulated after injury to the nervous system [11].

The voltage-gated sodium-channel type IX alpha subunit, known as Na_v_1.7 and encoded by the gene SCN9A [14], is located in peripheral neurons and plays an important role in the action potential of these cells. Na_v_1.7 is concentrated preferentially in rodent small diameter neurons [15]. The presence of Na_v_1.7 sodium channel has been demonstrated in sensory neurons in human dorsal root ganglia (DRG), including the majority of small and medium-sized neuronal cell bodies, which include the nociceptors [16]. The accumulation of this channel has been demonstrated within the neurite tips of DRG and trigeminal ganglion neurons in culture [17]. Electrophysiological studies show that Na_v_1.7 has a role in amplifying generator potentials [18].

The key role of Na_v_1.7 channels in pain conduction was confirmed in recent studies of clinical genetic disorders. Gain-of-function mutations in Na_v_1.7 have been shown to cause primary erythermalgia and familial rectal pain, recently renamed as paroxysmal extreme pain disorder [13,19], while loss-of-function mutations result in congenital insensitivity to pain [14]. Relatively few studies have evaluated expression of Na_v_1.7 in acquired clinical pain states, and even fewer within trigeminal pain states [2,20-25]. One recent study investigated pain within the orofacial pain region and showed an increase in Na_v_1.7 expression in pulpitis [22], while another study showed a decrease in Na_v_1.7 in the neuropathic orofacial pain condition trigeminal neuralgia (TN) [26]. The aim of our study was to investigate whether Na_v_1.7 has a role in BMS, considered a neuropathic orofacial pain disorder, and to compare this with dental pulpitis, an orofacial inflammatory pain condition.

## Methods

Patients scheduled for dental extraction, and those diagnosed with burning mouth syndrome (BMS), at Kings College London Dental Institute, London, were included in this study. Patients gave informed consent before participating in the study, which had approval from the North East London Ethics Committee. All patients were recruited sequentially, none of whom were taking any medication at the time of the study.

### Dental pulpitis

Seventeen permanent molar teeth were tested for positive neural vitality of the dental pulp one hour prior to extraction, using an electric pulp tester (analytic technology constant current at the mid-buccal surface of the tooth), and with ethyl chloride. The dental patients were divided into two groups, those with existing pain from the tooth (painful pulps; n = 5, with a mean age of 40.3 years [range 36-44 years]) and those with no history of or existing pain (controls; n = 12, with a mean age range of 37.3 years [range 23-51 years]). The gender distribution of the groups was M: F 1:1. All the dental pain in this study was attributable to pulpitis due to extensive dental caries of the molar tooth. Pulpitis was diagnosed by taking a full pain history and presence of pain on the day of extraction, vitality testing of the tooth and clinical radiography. The mean duration of pain was 2.9 weeks (range 0.5-8.0), and the indication for extraction of the non-painful teeth was pericoronitis.

All the teeth were removed by standard buccal approach under local or general anaesthesia. Subsequent to the extraction process (lasting less than 5 min), the teeth were sectioned vertically with a water-cooled drill and the pulp lifted out, and specimens immediately snap-frozen at -70°C.

### Burning mouth syndrome

Patients attending Kings College London Dental Institute with a diagnosis of burning mouth syndrome (BMS; n = 7) in accordance with the International Association for the Study of Pain (IASP) criteria [27], and those attending for wisdom tooth removal under local analgesia (controls; n = 10), were invited to join this study in accordance with the North East London Ethics Committee approval guidelines. Efforts were made to age and sex-match the BMS patients with controls; however, although the age ranges overlapped, BMS subjects were older than the controls. The mean age for the controls and the BMS patients was 40 years (range 16-79 years; M:F = 6:4) and 62 years (range 48-82 years; M:F = 5:5), respectively. The average duration of symptoms for the BMS group was 37.6 months. All patients were asked to report the degree of pain using the visual analogue scale (VAS) scoring system from 0 (no pain) to 10 (worst pain imaginable) prior to a lingual biopsy. The average pain score was 7.07 (range 5-10). All biopsies, including controls, were undertaken from the same site, on the right or left dorsal lingual mucosa lateral to the midline in the anterior third of the tongue, since all BMS patients had pain within this region.

Tongue punch biopsies were taken from BMS patients (disposable punch 3 × 3 mm Steifel CE 0120, Steifel Laboratories Ltd, Bucks, U.K.) under local anaesthesia. Control tongue punch biopsies were obtained from patients undergoing planned wisdom teeth surgery as a result of previous pericoronitis, which is localised mucosal inflammation around a partially erupted tooth. During recruitment of these control patients, a clinical history, examination and radiography were used to confirm this diagnosis. On the day of surgery, all control patients who attended for wisdom tooth removal were not in any pain (VAS = 0) as they were between episodes of pericoronitis. The control tongue biopsy was an additional procedure, which required no additional local anaesthesia. All tongue punch biopsies were immediately placed in liquid nitrogen, and subsequently transferred to -70°C storage until used for immunohistochemical analysis.

### Immunohistochemistry

Frozen specimens were embedded in OCT medium (RA Lamb, London, UK) and sections of 12 μm thaw-mounted onto glass slides pre-coated with poly-L-lysine. Sections were immersion-fixed in fresh 4% paraformaldehyde in phosphate buffered saline (PBS) for 30 minutes, then endogenous peroxidases blocked by incubation with alcoholic 0.3% hydrogen peroxide for a further 30 minutes. Sections were incubated overnight with a monoclonal antibody to the structural neuronal marker neurofilament (Clone 2F11, Dako, Cambridge, U.K., used at a final titre of 1:10, 000) and a polyclonal antibody against Na_v_1.7 (K241), whose specificity has been described by us previously [19,21]. Sites of primary antibody attachment were revealed using avidin-biotin peroxidase method (Vector Elite ABC method, Vectastain, Novacastra, Newcastle, UK). Preparations were counterstained in 1% w/v aqueous neutral red to visualise nuclei and photographed with an Olympus photomicroscope.

### Analysis

A visual observation method of analysis for the evaluation of Na_v _1.7 in the subodontoblast plexus and submucosal region of tongue biopsies was adopted. This was in accordance with previously described methods [28,29]. A visual grading scale of intensity ranging between 0-3 (0 = nil, 1 = weak, 2 = moderate and 3 = strong immunoreactivity) was used. The mean values of readings obtained by two independent observers, each blinded, were used for final analysis. Computerised image analysis was also performed to quantify Na_v _1.7 immunoreactive area in the subodontoblast plexus of dental pulp, and submucosal region of tongue biopsies. Images were captured using an Olympus DP70 camera mounted to an Olympus BX50 microscope and analysed using analySIS (version 5.0). Positive Na_v _1.7 and neurofilaments immunostaining was highlighted by setting the grey-level detection limits to threshold and the area of highlighted immunoreactivity obtained as% area of the field scanned. Five random fields per tissue section were scanned and the mean value used in subsequent statistical analysis. The Mann-Whitney test was used for statistical analysis (P values < 0.05 were considered statistically significant). The ratio between Na_v _1.7 and neurofilaments in serial tissue sections was calculated and used to compare the results from the different groups. The Mann Whitney test was used to compare ratios between groups; *P *values less than 0.05 were considered significant.

## Results

**Figure 1:**
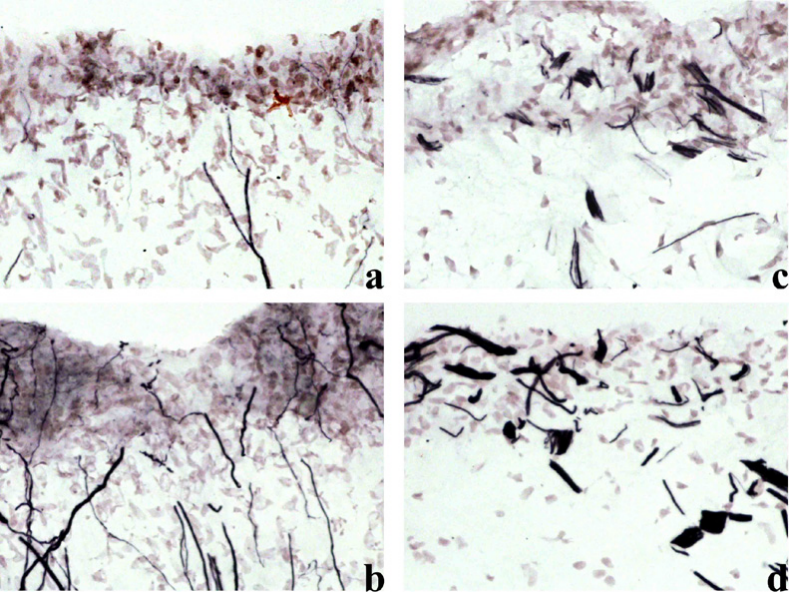
**Immunoreactive nerve fibres in non-painful (left column) and painful (right column) human tooth pulp sections, within the subodontoblastic plexus region**. Staining with antibodies to Na_v_1.7 (Figures 1a and 1c) and neurofilament cocktail (Figures 1b and 1d). Magnification × 40.

**Figure 2:**
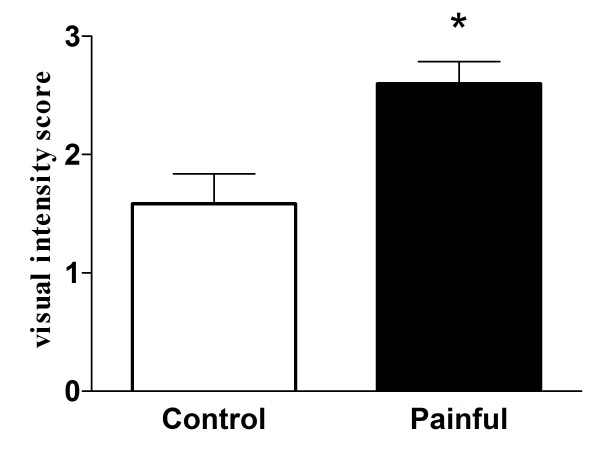
Visual intensity score of Na_v_1. 7 in tooth pulp (mean ± SEM). * P < 0.005.

**Figure 3:**
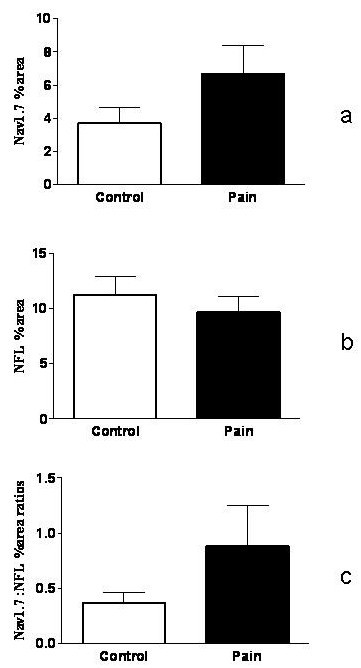
**Image analysis of control and painful tooth pulp using antibodies to Na_v_1.7 (Figure 3a), neurofilament (Figure 3b) and expressing the results as a ratio of Na_v_1.7:NFL (Figure 3c)**.

Figure 1 shows the presence of large numbers of nerve fibres within human tooth pulp that were immunoreactive for neurofilaments (Figure 1b, d). A subset of nerve fibres was also immunostained with the Na_v_1.7 antibody (Figure 1a, c) in both non-painful and painful pulp groups. There was a significant increase in the visual intensity score for Na_v_1.7 in the painful group compared to controls (Figure 2). By image analysis, there was a trend for an increase of the Na_v_1.7 immunoreactive area in the painful group, but this was not statistically significant (Figure 3a). There was no difference between the neurofilament staining in these groups (Figure 3b), and when the results were expressed as a ratio of the neurofilament% area, there was also a strong trend for an increase of Na_v_1.7 in the painful group (Figure 3c).

**Figure 4:**
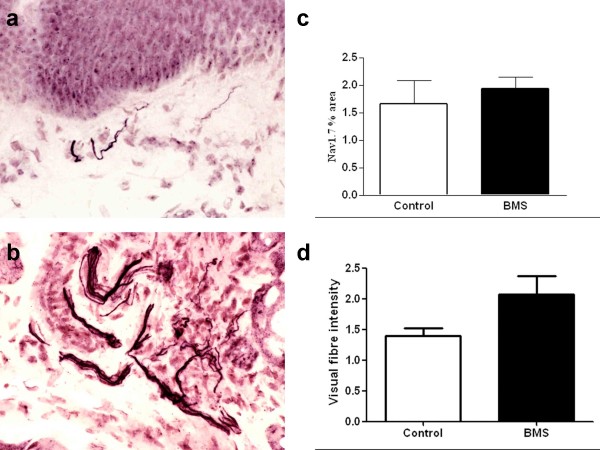
**Immunoreactive Na_v_1.7 nerve fibres in control tongue (Figure 4a) and burning mouth syndrome (Figure 4b), magnification × 40**. The bar charts (Figure 4c) show the image analysis and visual intensity scores (Figure 4d) of the Na_v_1.7 fibres in tongue (Mean ± SEM).

There was an abundance of Na_v_1.7 immunoreactive fibres in the sub-mucosal layer of both control (Figure 4a) and BMS tongue biopsies (Figure 4b). No significant difference was observed between BMS and control biopsies with image analysis (Figure 4c). There was a trend for an increase in the visual intensity score for Na_v_1.7 in the BMS compared to controls, but this was not statistically significant (Figure 4d).

## Discussion

The role of ion channels has been implicated in subtypes of pain, such as neuropathic and inflammatory pain [11]. Our study aimed to investigate and compare the expression of Na_v_1.7 sodium channel within two orofacial pain disorders, an inflammatory pain condition dental pulpitis, and burning mouth syndrome (BMS), considered a neuropathic condition.

This study showed a significant increase in the visual intensity levels of Na_v_1.7 immunoreactivity within painful dental pulp, in accord with a previous study by Luo et al. (2008) [22]. The latter reported an increase within the axonal bundles [22], and in our study we observed increases within the subodontoblastic plexus. With over 900 axons known to enter the average human premolar tooth, less than half of these fibres that innervate the dental pulp (40%) terminate in dentinal tubules, close to the odontoblast process. The remaining fibres form the subodontoblastic plexus in the coronal aspect of the pulp tissue [2]. Our study has helped to localise the increased expression of Na_v_1.7 to the subodontoblastic plexus within painful pulp. These findings indicate a possible involvement of Na_v_1.7 in toothache, and also suggest a potential role in human inflammatory pain conditions. The inflammatory mediators within pulp may up-regulate the expression of Na_v_1.7, as may micro-organisms within the oral cavity by causing inflammation. It is unlikely that extracting teeth, or taking tongue biopsies, under local or general anaesthesia will affect Na_v _channel expression; the acute effects of anaesthetics would not be expected to alter Na_v _channel levels in the short term, as Na_v _channels are synthesized in cell bodies and axonally transport to the nerve terminals where they are inserted into membranes, a process with a turnover of several days.

Previous studies have demonstrated the role of other sodium channel isoforms within painful pulps. Renton et al. (2005) showed a significant increase in Na_v_1.8 fibres as a proportion of neurofilament positive fibres within painful dental pulp specimens [2]. Wells et al. (2007) reported a significant increase in the immunoreactivity of Na_v_1.9 channels in the axons of painful teeth compared to control teeth with no pain [25]. Padilla et al. (2007) also studied the expression of Na_v_1.9, within rat and mouse trigeminal ganglion nerve endings [24]. Na_v_1.9 was expressed in trigeminal ganglion neuronal somata (small and medium sized), and also along trigeminal afferent fibres and the terminal branches within lip skin and dental pulp.

The role of sodium channels has further been demonstrated in other inflammatory pain conditions. Strickland et al. (2008) investigated the changes in expression of Na_v_1.7, Na_v_1.8 and Na_v_1.9 sodium channels in the rat model and showed that the dorsal root ganglia innervating the knee joint had increased expression of all three subtypes up to 28 days after the initial insult [30]. Our previous study demonstrated an increase of sodium channels Na_v_1.7, Na_v_1.8 and Na_v_1.9 in hypersensitivity states of nasal mucosa in patients with allergic and non-allergic rhinitis [21]. These studies support a potential role of these sodium channels in chronic inflammatory pain and hypersensitivity [30].

Burning mouth syndrome (BMS) is defined as a distinct neuropathic orofacial pain condition affecting the oral mucosa according to the International Association for the Study of Pain (IASP). The classification of BMS as a neuropathic pain condition is supported by several previous studies which demonstrate the loss of intra-epithelial nerve fibres, and evidence of small fibre neuropathic changes [31,32]. The BMS patients who participated in this study were recruited in accordance to the International Association for the Study of Pain (IASP) in order to minimise any heterogeneity [27]. BMS patients reported moderate to severe pain intensity, which was similar to previous studies [7,8]. The expression of Na_v_1.7 sodium channel in sub-epithelial nerve fibres did not change significantly in BMS in this study. While efforts were made to match the BMS patients with controls and the age ranges overlap, the BMS subjects were older than the controls, and this needs to be taken into consideration. Our previous work also did not show a significant change in another sodium channel isoform Na_v_1.8 in BMS, whereas the levels of the heat and capsaicin receptor, transient receptor potential Vanilloid (TRPV1), were increased [31].

As we did not observe a significant change in TRPV1 immunoreactivity levels in painful dental pulpitis compared to non-painful tooth pulp [33], there appears to be differential expression of sodium and TRP channels in trigeminal pain disorders - further studies are required to confirm the increase of Na_v_1.7, Na_v_1.8 and Na_v_1.9 in inflammatory and TRPV1 in neuropathic trigeminal pain states. A recent investigation in trigeminal neuralgia, a neuropathic pain condition, demonstrated that Na_v_1.7 levels were decreased. Na_v_1.7 thus appears more likely to be involved in acquired inflammatory pain, rather than neuropathic pain, in trigeminal disorders [26].

The development of novel Na_v_1.7 selective blockers remains a challenge, and a major pharmaceutical goal [34] - studies such as this may identify suitable patient cohorts for clinical trials with selective sodium channel antagonists.

## Conclusions

The results of this study suggest that the Na_v_1.7 sodium channel may play a role in inflammatory dental pain. Clinical trials with selective Na_v_1.7 channel blockers may prioritise dental pulp pain rather than burning mouth syndrome.

## Competing interests

The authors declare that they have no competing interests.

## Authors' contributions

KB and TR performed all the surgical procedures, extracted the tooth pulp, carried out all mucosal biopsies and assisted with interpretation of the data. YY performed the immunohistochemistry studies, and participated in the analysis of data and preparation of the manuscript. ZY assisted in drafting the manuscript. TR and PA conceived and coordinated the study, assisted with interpretation of results and helped write the manuscript. KB completed the writing of the manuscript. All authors read and approved the manuscript.

## References

[B1] Lipton JA Ship JA Larach-Robinson D Estimated prevalence and distribution of reported orofacial pain in the United States J Am Dent Assoc 1993 124 10 115 121 8409001 10.14219/jada.archive.1993.0200

[B2] Renton T Yiangou Y Tate S Bountra C Anand P Sodium channel Nav1 8 immunoreactivity in painful human dental pulp BMC Oral Health 2005 5 1 5 10.1186/1472-6831-5-5 16001984 PMC1183220

[B3] Hildebrand C Fried K Tuisku F Johansson CS Teeth and tooth nerves Prog Neurobiol 1995 45 165 222 10.1016/0301-0082(94)00045-J 7777672

[B4] Drenth JP Waxman SG Mutations in sodium-channel gene SCN9A cause a spectrum of human genetic pain disorders J Clin Invest 2007 117 12 3603 9 10.1172/JCI33297 18060017 PMC2096434

[B5] Fried K Hildebrand C Pulpal axons in developing, mature, and aging feline permanent incisors A study by electron microscopy J Comp Neurol 1981 203 23 36 10.1002/cne.902030104 7309916

[B6] Närhi M The neurophysiology of the teeth Dent Clin North Am 1990 34 3 439 48 2197120

[B7] Buchanan J Zakrzewska J Burning mouth syndrome Clin Evid 2004 12 1899 905 15865757

[B8] Grushka M Sessle BJ Miller R Pain and personality profiles in burning mouth syndrome Pain 1987 28 169 84 10.1016/0304-3959(87)90114-X 3822501

[B9] Grushka M Burning mouth syndrome: pain disorder remains difficult to treat Ont Dent 1987 64 26 30 3470697

[B10] Granot M Nagler RM Association between regional idiopathic neuropathy and salivary involvement as the possible mechanism for oral sensory complaints J Pain 2005 6 581 7 10.1016/j.jpain.2005.03.010 16139777

[B11] Rogers M Tang L Madge DJ Stevens EB The role of sodium channels in neuropathic pain Semin Cell Dev Biol 2006 17 5 571 81 10.1016/j.semcdb.2006.10.009 17123847

[B12] Dib-Hajj SD Cummins TR Black JA Waxman SG From genes to pain: Nav 1.7 and human pain disorders Trends Neurosci 2007 30 11 555 63 10.1016/j.tins.2007.08.004 17950472

[B13] Drenth JP Te RH Morsche Mansour S Mortimer PS Primary erythermalgia as a sodium channelopathy: screening for SCN9A mutations: exclusion of a causal role of SCN10A and SCN11A Arch Dermatol 2008 144 3 389 91 10.1001/archderm.144.3.320 18347287

[B14] Cox JJ Reimann F Nicholas AK Thornton G Roberts E Springell K Karbani G Jafri H Mannan J Raashid Y Al-Gazali L Hamamy H Valente EM Gorman S Williams R McHale DP Wood JN Gribble FM Woods CG An SCN9A channelopathy causes congenital inability to experience pain Nature 2006 444 7121 894 898 10.1038/nature05413 17167479 PMC7212082

[B15] Djouhri L Newton R Levinson SR Berry GM Carruthers B Lawson SN Sensory and electrophysiological properties of guinea-pig sensory neurones expressing Nav1 7 (PN1) Na^+^channel alpha subunit protein J Physiol 2003 546 Pt2 565 76 10.1113/jphysiol.2002.026559 12527742 PMC2342520

[B16] Coward K Aitken A Powell A Plumpton C Birch R Tate S Bountra C Anand P Plasticity of TTX-sensitive sodium channels PN1 and brain III in injured human nerves Neuroreport 2001 12 495 500 10.1097/00001756-200103050-00014 11234752

[B17] Toledo-Aral JJ Moss BL He ZJ Koszowski AG Whisenand T Levinson SR Wolf JJ Silos-Santiago I Halegoua S Mandel G Identification of PN1, a predominant voltage-dependent sodium channel expressed principally in peripheral neurons Proc Natl Acad Sci USA 1997 94 1527 1532 10.1073/pnas.94.4.1527 9037087 PMC19825

[B18] Cummins TR Howe JR Waxman SG Slow closed-state inactivation: a novel mechanism underlying ramp currents in cells expressing the hNE/PN1 sodium channel J Neurosci 1998 18 9607 9619 9822722 10.1523/JNEUROSCI.18-23-09607.1998 PMC6793269

[B19] Yiangou Y Facer P Chessell IP Bountra C Chan C Fertleman C Smith V Anand P Voltage-gated ion channel Nav1 7 innervation in patients with idiopathic rectal hypersensitivity and paroxysmal extreme pain disorder (familial rectal pain) Neurosci Lett 2007 427 2 77 82 10.1016/j.neulet.2007.09.027 17928139

[B20] Henry MA Freking AR Johnson LR Levinson SR Sodium channel Nav 1.6 accumulates at the site of infraorbital nerve injury BMC Neurosci 2007 8 56 10.1186/1471-2202-8-56 17662136 PMC1941742

[B21] Keh M Simpson K Sandhu G Saleh H Anand P Increased nerve fiber expression of sensory sodium channels Nav 17, 1.8 and 1.9 in rhinitis Laryngoscope 2008 118 573 9 10.1097/MLG.0b013e3181625d5a 18197135

[B22] Luo S Perry GM Levinson SR Henry MA Nav 1.7 expression is increased in painful human dental pulp Mol Pain 2008 21 4 16 10.1186/1744-8069-4-16 PMC2377237 18426592

[B23] Bird EV Robinson PP Boissonade FM Na(v)1.7 sodium channel expression in human lingual nerve neuromas Arch Oral Biol 2007 52 5 494 502 10.1016/j.archoralbio.2006.11.011 17210118

[B24] Padilla F Couble ML Coste B Maingret F Clerc N Crest M Ritter AM Magloire H Delmas P Expression and localization of the Nav 1.9 sodium channel in enteric neurons and in trigeminal sensory endings: implication for intestinal reflex function and orofacial pain Mol Cell Neurosci 2007 35 1 138 52 10.1016/j.mcn.2007.02.008 17363266

[B25] Wells JE Bingham V Rowland KC Hatton J Expression of Nav1.9 channels in human dental pulp and trigeminal ganglion J Endod 2007 33 10 1172 6 10.1016/j.joen.2007.05.023 17889684

[B26] Siqueira SR Alves B Malpartida HM Teixeira MJ Siqueira JT Abnormal expression of voltage-gated sodium channels Nav 17, Nav1.3 and Nav1.8 in trigeminal neuralgia Neuroscience 2009 164 573 7 10.1016/j.neuroscience.2009.08.037 19699781

[B27] Zakrzewska JM Glenny AM Forsell H Interventions for the treatment of burning mouth syndrome Cochrane Database Syst Rev 2001 3 CD002779 11687027 10.1002/14651858.CD002779

[B28] Brady CM Apostolidis A Yiangou Y Baecker PA Ford AP Freeman A Jacques TS Fowler CJ Anand P P2 × 3-immunoreactive nerve fibres in neurogenic detrusor overactivity and the effect of intravesical resiniferatoxin Eur Urol 2004 46 247 253 10.1016/j.eururo.2003.12.017 15245821

[B29] Brady CM Apostolidis AN Harper M Yiangou Y Beckett A Jacques TS Freeman A Scaravilli F Fowler CJ Anand P Parallel changes in bladder suburothelial vanilloid receptor TRPV1 and pan-neuronal marker PGP9 5 immunoreactivity in patients with neurogenic detrusor overactivity after intravesical resiniferatoxin treatment BJU Int 2004 93 770 776 10.1111/j.1464-410X.2003.04722.x 15049988

[B30] Strickland IT Martindale JC Woodhams PL Reeve AJ Chessell IP McQueen DS Changes in the expression of Nav1 7, Nav1.8 and Nav1.9 in a distant population of dorsal root ganglia innervating the rat knee joint in a model of chronic inflammatory joint pain Eur J Pain 2008 12 564 72 10.1016/j.ejpain.2007.09.001 17950013

[B31] Yilmaz Z Renton T Yiangou Y Zakrzewska J Chessell IP Bountra C Anand P Burning mouth syndrome as a trigeminal small fibre neuropathy: Increased heat and capsaicin receptor TRPV1 in nerve fibres correlates with pain score J Clin Neurosci 2007 14 9 864 71 10.1016/j.jocn.2006.09.002 17582772

[B32] Suarez V Guntinas-Lichius O Streppel M Ingorokva S Grosheva M Neiss WF Angelov DN Klimaschewski L The axotomy-induced neuropeptides galanin and pituitary adenylate cyclase-activating peptide promote axonal sprouting of primary afferent and cranial motor neurones Eur J Neurosci 2006 24 1555 64 10.1111/j.1460-9568.2006.05029.x 17004919

[B33] Renton T Yiangou Y Baecker PA Ford AP Anand P Capsaicin receptor VR1 and ATP purinoceptor P2 × 3 in painful and nonpainful human tooth pulp J Orofac Pain 2003 17 3 245 50 14520770

[B34] Gold MS Sodium channels and pain therapy Curr Opin Anaesthesiol 2000 13 5 565 72 10.1097/00001503-200010000-00014 17016359

